# Phenotyping of Arrhythmia-induced Cardiomyopathy: From Bench to Bedside and Back

**DOI:** 10.1007/s11897-026-00788-3

**Published:** 2026-10-27

**Authors:** Thomas Körtl, Johannes Ruppert, Samuel Sossalla

**Affiliations:** 1 Medical Clinic I, Cardiology and Angiology, University Hospital of Giessen and Marburg, Giessen, Germany; 2 Institute for Experimental and Translational Cardiology, Justus-Liebig-Universität Giessen, Giessen, Germany; 3 Department of Cardiology at Kerckhoff Heart and Lung Center, Bad Nauheim, Germany; 4 Excellence Cluster Cardiopulmonary Institute (CPI), Giessen, Germany

**Keywords:** Arrhythmia-induced cardiomyopathy, Atrial fibrillation, Heart failure, Rhythm control therapy, Phenotyping

## Abstract

**Purpose of Review:**

Arrhythmia-induced cardiomyopathy (AIC) is an increasingly recognized and potentially reversible cause or modifier of heart failure. This review summarizes experimental and clinical evidence with a focus on atrial fibrillation (AFib)-induced left ventricular systolic dysfunction.

**Recent Findings:**

Recent studies demonstrate that AIC extends beyond tachycardia alone and involves rhythm irregularity, loss of atrioventricular synchrony, altered calcium handling, oxidative stress, mitochondrial dysfunction and substrate vulnerability. Clinical trials demonstrate the superiority of rhythm control therapy by catheter ablation in patients with AFib and heart failure. Emerging data illustrate the importance of phenotyping of AIC according to myocardial substrate and individual ventricular response to AFib.

**Summary:**

AIC should be considered as a heterogeneous spectrum. Refined phenotyping may improve prediction of recovery and guide specific and individual therapy.

## Introduction

Arrhythmia-induced cardiomyopathy (AIC) has evolved from an early experimental observation into a clinically relevant and increasingly recognized heart failure phenotype. Rapid-pacing animal studies demonstrated that sustained tachycardia induces progressive ventricular dilation, systolic dysfunction, neurohormonal activation and heart failure, while termination of pacing leads to functional recovery [1, 3]. These findings provided the foundation for the concept of tachycardia-induced cardiomyopathy.

However, the modern concept of AIC extends beyond tachycardia alone. AIC is defined as a potentially reversible myocardial dysfunction in which an arrhythmia acts as the primary cause or an aggravating factor of left ventricular (LV) systolic dysfunction (LVSD). Rhythm disorders that trigger AIC include atrial fibrillation (AFib), atrial flutter (AFlut), atrial tachycardia (AT), atrioventricular (nodal) re-entry tachycardia (AV(N)RT), ventricular tachycardia (VT) and frequent premature ventricular contractions (PVC) [4, 5].

The incidence and prevalence of AIC remain difficult to determine because the diagnosis is usually established retrospectively and depends on recovery of LV ejection fraction (LVEF) after effective rate or rather rhythm control therapy. An additional problem in this context is that in many cases, it is also difficult to determine if an arrhythmia is the underlying cause or consequence of heart failure [6]. A recent systematic review and meta-analysis including 26 studies and 7331 patients estimated the overall prevalence of AIC at approximately 8% but found a substantially higher prevalence of 59% in cohorts with LVSD. Besides, prevalence varied according to the underlying arrhythmia and definition of AIC [7]. In the DECAAF II post hoc trial, 72 of 119 patients (61%) with persistent AFib and LVSD fulfilled AIC criteria after AFib ablation [8]. In a trial from our own group, 82% of patients with newly diagnosed, otherwise unexplained LVSD and concomitant tachyarrhythmia (AFib and AFlut) were classified as having AIC 6 months after rhythm restoration [9]. Among all arrhythmias, AFib is the most important clinical driver of AIC. This is supported by landmark trials showing that AFib-mediated LVSD is common and recovery of LVEF is strongly dependent on effective rhythm control [10, 12].

Experimental work provides the mechanistic bridge between arrhythmia burden and ventricular dysfunction. Rapid-pacing models show that sustained tachycardia induces impaired excitation-contraction coupling, altered calcium handling, mitochondrial dysfunction, oxidative stress, extracellular matrix remodeling and ventricular dilatation [13, 15]. PVC-specific models add another mechanistic layer by establishing that dyssynchronous ventricular activation leads to cardiomyopathy even without sustained tachycardia, whereas AFib-related data indicate that rhythm irregularity and AFib burden promote distinct ventricular cellular and molecular remodeling [16, 19].

This review will integrate clinical and experimental evidence on AIC with a particular focus on AFib-mediated LVSD. A useful phenotyping framework will be proposed which allows for better differentiation among individual patients with AIC.

## Experimental Evidence in Arrhythmia-induced Cardiomyopathy

The mechanistic understanding of AIC has been primarily derived from animal models. These models have provided specific platforms in which individual arrhythmic components such as sustained rapid activation, irregular ventricular response or loss of atrioventricular synchrony can be studied [4]. There are several different approaches to examine the influence of rhythm disorders in animals. These include long-term right ventricular (RV) pacing after atrioventricular node ablation, overdrive RV pacing to induce ventricular dysfunction and arrhythmias as well as ventricular pacing models that secondarily promote atrial dilatation, fibrosis and AFib [20].

Contemporary preclinical models address not only if tachycardia can induce heart failure, but also how different stressors like stimulation frequency or ectopic burden remodel the myocardium. Recent work has emphasized that pacing protocols must be carefully optimized to balance reproducibility with animal welfare and model stability. In a study with pigs, gradual ventricular pacing up to 200 bpm over four weeks led to a reproducible heart failure phenotype with impaired cardiac function and reduced exercise capacity, while subsequent pacing with lower heart rates helped to maintain dysfunction without excessive decompensation [21].

Another recent study suggested that tachycardia-induced myocardial dysfunction is not merely a secondary consequence of impaired pump capability but involves a distinct metabolic phenotype. In a combined rabbit tachypacing and human induced pluripotent stem cell-derived cardiomyocyte (iPSC-CM) model, tachycardia exposure shifted myocardial metabolism away from a preserved energetic state toward mitochondrial disorganization, redox imbalances and depletion of tricarboxylic acid cycle (TCA) metabolites, while development of fibrosis was not a dominant feature. These findings support the concept that AIC differs from other forms of cardiomyopathies [22].

Complementary mechanistic data from a murine model in which chronic tachycardia and cardiac work overload was induced by isoproterenol strengthen the role of mitochondria in the development of heart failure. Elevated mitochondrial reactive oxygen species (ROS) were accompanied by remodeling of the mitochondrial cristae and linked to activation of the mitochondrial protease OMA1, whereas absence of this protease prevented cardiomyocyte death. Although the authors did not use a classical tachypacing model, these findings provide a mechanistic link between sustained cardiac stress and mitochondrial injury pathways that may be relevant to AIC [23].

However, animal models also have important translational limitations. Many tachypacing protocols rely on chronic RV pacing, which itself triggers ventricular dysfunction and heart failure [24]. Species specific differences exist between animal models and human biology [25], which further limit direct extrapolation of findings from preclinical models to human AIC.

**Fig. 1:**
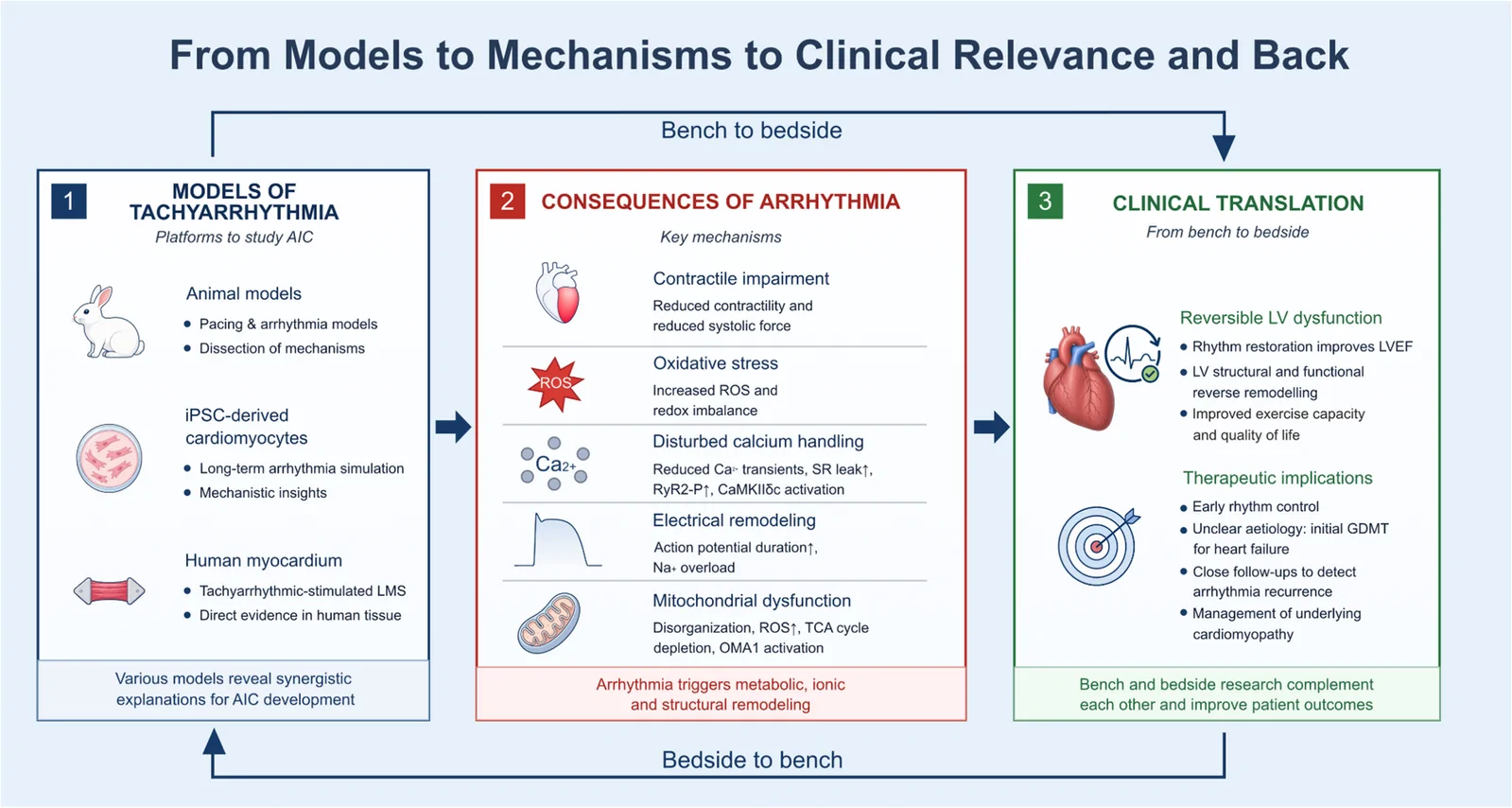
Translational perspective of AIC. There are several different platforms to investigate the key mechanisms of arrhythmia, including animal models, iPSC-CM and primary human myocardium. Evidence from preclinical research is used to better understand the translational relevance of arrhythmia-associated remodeling and to improve experimental models. AFib=atrial fibrillation, AIC=arrhythmia-induced cardiomyopathy, CaMKII IIδc=calcium/calmodulin-dependent protein kinase IIδc, iPSC-CM=human induced pluripotent stem cell-derived cardiomyocyte, LV=left ventricular, LVEF=left ventricular ejection fraction, ROS= reactive oxygen species, RyR2-P=ryanodine receptor 2 phosphorylation, TCA=tricarboxylic acid cycle

Direct experimental evidence for AIC in human myocardium remains limited, but a translation study from our group demonstrated that even normofrequent AFib causes several deleterious alterations in the LV [18]. We investigated myocardium from patients with preserved LV function and compared tissue from individuals in sinus rhythm with those from patients with rate controlled AFib. Despite an absence of ventricular fibrosis, tissue from AFib patients was characterized by altered calcium homeostasis with a reduction of calcium transient amplitudes and an increase in diastolic sarcoplasmic reticulum leak. In addition, prolongation of cardiac action potentials and elevation of cytosolic sodium concentration was observed after AFib simulation in an iPSC-CM model. Mechanistically, ROS were higher in AFib which was accompanied by a subsequent oxidation and activation of calcium/calmodulin-dependent protein kinase IIδc (CaMKII IIδc) as well as an increased ryanodine receptor 2 phosphorylation (RyR2-P) [18]. Further work showed that not only the arrhythmia per se, but rather the amount of AFib burden is crucial for ventricular cellular remodeling, with higher AFib exposure inducing reduced calcium transient amplitude, prolonged action potentials and impaired sarcomere regularity [19]. More recent human data demonstrated that pre-existing heart failure increases susceptibility to AFib-induced ventricular dysfunction. AFib simulation rapidly impaired contractile function in failing, but not in non-failing myocardium and induced adverse electrophysiological remodeling in iPSC-CM derived cardiomyocytes derived from patients with dilated cardiomyopathy [26] (Fig. 1).

## Definition and Key Clinical Evidence for Arrhythmia-induced Cardiomyopathy

### Definition and Terminology

**Fig. 2:**
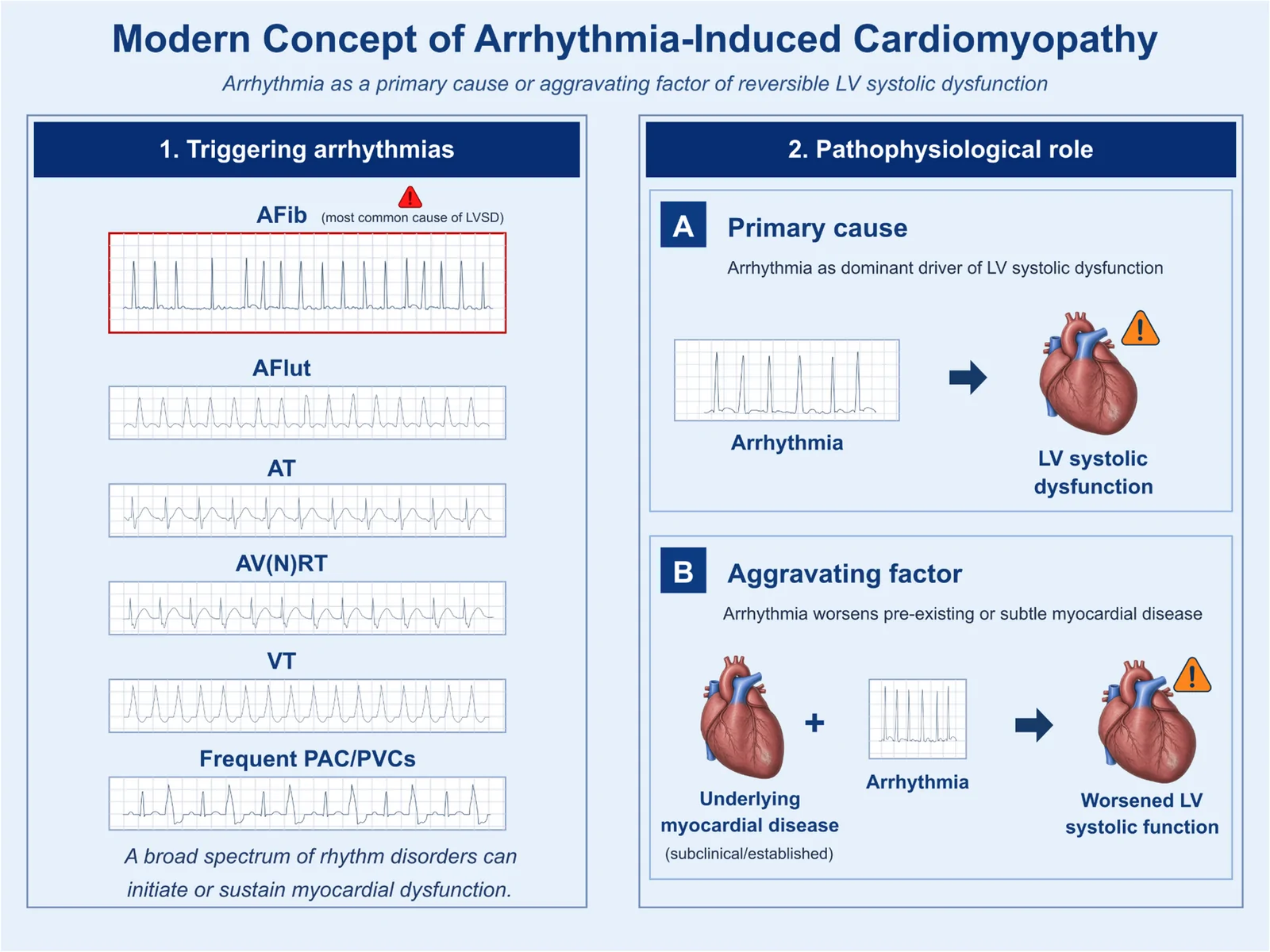
Definition of AIC. Multiple supraventricular and ventricular rhythm disorders can trigger reversible LVSD, with AFib being the most common type of arrhythmia. AIC either presents as a “pure” form which is arrhythmia-driven or a “superimposed” form, in which the arrhythmia represents an aggravating factor for a pre-existing heart failure. AFib=atrial fibrillation, AFlut=atrial flutter, AIC=arrhythmia-induced cardiomyopathy, AT=atrial tachycardia, AV(N)RT= atrioventricular (nodal) re-entry tachycardia, LV=left ventricular, LVSD=left ventricular systolic dysfunction, PVC=premature ventricular contraction, VT=ventricular tachycardia

AIC can be defined as a potentially reversible syndrome of LVSD in which tachyarrhythmia or a high ectopic burden cause, sustain or aggravate myocardial dysfunction. A “pure” form, in which the arrhythmia is the sole driver of LV dysfunction, can be distinguished from a “mediated/superimposed” form, in which the arrhythmia worsens a pre-existing heart failure [6] (Fig. 2).

Although AIC is increasingly recognized as a driver of LVSD and research on this topic intensifies, there is still no universally accepted diagnostic definition of AIC. The 2024 EHRA Scientific Initiatives Committee survey showed marked heterogeneity in clinical practice and specifically highlighted uncertainty regarding how much LVEF deterioration or recovery is required to label a case as AIC [5]. This lack of standardization also explains why reported prevalence varies across cohorts. For clinical studies, three recovery thresholds are most often used. In some cohorts, recovery is defined as an LVEF increase of ≥ 10 or ≥ 15% points, while others use complete recovery of LV function as the defining criterion, typically requiring a final LVEF of ≥ 50%. Trial entry cutoffs are even more heterogenous, with an LVEF range from < 35% to < 55% [7].

We favor a combined approach that defines AIC as an absolute improvement of ≥ 15% points or ≥ 10% points in patients achieving a final LVEF ≥ 50%, as previously applied in our prospective AIC cohort [9].

### Atrial Fibrillation and Heart Failure

AFib and heart failure frequently coexist and are linked by a bidirectional relationship. AFib is present in more than half of patients with heart failure, whereas more than one third of patients with AFib have concomitant heart failure. Of note, AFib affects patients across the entire heart failure spectrum, including both heart failure with reduced and preserved ejection fraction (HFrEF and HFpEF) [27]. Regardless of which condition occurs first, patients with AFib and heart failure have worse outcomes with higher rates of hospitalization, stroke and mortality. AFib is also associated with poorer health-related quality of life in HFrEF and HFpEF [28, 29].

The close interaction between AFib and heart failure has direct therapeutic implications. The current 2024 ESC Guidelines for the management of AFib endorse catheter ablation in patients with AFib and HFrEF for achieving LVEF recovery, particularly when AIC is suspected (class I recommendation), and state that ablation should be considered in selected patients with AFib and HFrEF to reduce heart failure hospitalization and improve survival (class IIa recommendation). This guideline position is largely based on randomized clinical trials showing the superiority of interventional rhythm control in patients with AFib and HFrEF [30].

Among all AFib ablation studies, CAMERA-MRI is arguably one of the most conceptually important for AIC, because it enrolled patients with AFib and otherwise unexplained LVSD. In this trial, catheter ablation led to a much larger improvement in LVEF that medical rate control at 6 months (18% vs. 4%), and the absence of late gadolinium enhancement (LGE) on cardiac magnetic resonance imaging (MRI) was associated with greater recovery and a higher likelihood of LVEF normalization [10]. In addition, long-term follow up over 4 years demonstrated that catheter ablation led to greater improvement in LVEF, with the most pronounced effects observed in patients without ventricular fibrosis [31]. CAMERA-MRI II extended these findings to patients with AFib, LVSD and pre-existing ventricular fibrosis (LGE ≥5%). In this population, catheter ablation improved LVEF to a greater extent that medical rate control after 12 months of follow-up and was associated with better functional capacity, lower NT-proBNP levels and improved quality of life. However, LVEF recovery was reduced with increasing LGE burden, which suggest that fibrosis modifies, but does not eliminate the potential for reverse remodeling after rhythm control [32]. Collectively, these studies highlight that restoration of sinus rhythm is substantially more important than rate control alone, underscoring the central role of AFib itself in the pathogenesis of LVSD, which carries important implications for both mechanistic understanding and therapeutic decision-making. The CASTLE-AF trial investigated hard clinical endpoints and enrolled symptomatic AFib patients with LVEF ≤35% and implantable device. Catheter ablation reduced the primary composite endpoint of death or heart failure hospitalizations (28.5% vs. 44.6%) compared to standard medical therapy [11]. In a more advanced heart failure population, CASTLE-HTx showed that in patients with AFib and end-stage heart failure who were evaluated for transplant, catheter ablation in addition to guideline-directed medical therapy (GDMT) was superior in reducing the composite endpoint of all-cause death, LV assistant device implantation or urgent transplantation compared with medical therapy alone [33]. The EAST-AFNET 4 trial broadens the perspective beyond HFrEF and AIC specific cohorts. In patients with recently diagnosed AFib, those who were referred to early rhythm control with antiarrhythmic drugs or ablation experienced lower rate of adverse cardiovascular events than those who were assigned to usual care [34]. Although individual trials consistently support rhythm control in patients with AFib and HFrEF, they differ in study population, heart failure severity and treatment regime. A stratified pooled analysis of 11 randomized trials with 3598 patients by Chen et al. showed that clinical benefit was mainly driven by catheter ablation rather than antiarrhythmic drug-based therapy, with ablation being associated with lower mortality, fewer hospitalizations, greater LVEF improvement and better quality of life [35].

Compared with HFrEF, evidence guiding rhythm-control strategies in patients with AFib and HFpEF remains substantially less robust. This is reflected in the 2024 ESC Guidelines for the management of AFib, which do not issue a specific statement regarding catheter ablation of AFib in HFpEF but merely recommend dronedarone for long-term rhythm control to prevent arrhythmia relapse and AFib progression in patients with HFpEF [30]. Nevertheless, emerging data suggests that catheter ablation may also be beneficial in HFpEF and AFib. A post hoc analysis of the CABANA trial which randomized patients at risk for stroke to catheter ablation or drug therapy demonstrated that interventional arrhythmia treatment was associated with a lower risk for death or hospitalizations in patients who had a high likelihood of HFpEF according to the H_2_FPEF score [36]. The ongoing CABA-HFPEF study that randomizes AFib patients with HFpEF or heart failure with mildly reduced ejection fraction to early catheter ablation or usual care will determine the role of catheter ablation in this cohort [37].

Taken together, the available clinical evidence indicates that AFib acts as a modifiable driver of ventricular dysfunction, symptoms and adverse outcomes in heart failure. Importantly, the therapeutic relevance of catheter ablation does not appear to be restricted to patients with a “pure” form of AFib-mediated AIC. In patients with pre-existing myocardial disease, ventricular fibrosis or advanced heart failure, rhythm control by catheter ablation also improves LVEF, reduces heart failure events and enhances functional status.

### Hemodynamic Implications of Rhythm Restoration in Atrial Fibrillation

From a hemodynamic perspective, AFib impairs cardiac performance through several mechanisms that extend beyond tachycardia alone. The loss of coordinated atrial contraction abolishes the “atrial kick”, which normally contributes to ventricular filling, while irregular beat-to-beat intervals further reduce the efficiency of ventricular filling and forward flow [38]. Very early data after AFib ablation suggest that restoration of sinus rhythm triggers rapid functional improvement. In a prospective study of 117 patients undergoing catheter ablation, direct biatrial pressure measurements and echocardiography before and after 48 h after ablation showed that mean left atrial pressure increased from 12.2±5.4 mmHg to 16.0±5.8 mmHg and mean right atrial pressure from 8.2±4.2 mmHg to 10.8±4.9 mmHg, likely reflecting acute procedural and volume related effects. At the same time, biatrial function and LV global longitudinal strain improved, and RV functional parameters including tricuspid annular plane systolic excursion (TAPSE) as well as RV fraction area change increased, whereas chamber volumes remained unchanged. Thus, recovery may begin very early after rhythm restoration, although this early phase may be accompanied by transient LV diastolic stunning and elevated atrial pressures [39].

In patients with HFpEF, the STALL AF-HFpEF trial showed that catheter ablation was associated with a reduction in peak exercise pulmonary capillary wedge pressure (PCWP), lower BNP levels and better quality of life among patients who remained in sinus rhythm after ablation [40]. Similar results were demonstrated in a randomized study including patients with AFib and invasively confirmed HFpEF who were treated with catheter ablation or medical therapy. After 6 months, patients in the ablation group had reduced PCWP and improved exercise capacity as well as quality of life. Importantly, 50% of patients referred to ablation no longer fulfilled invasive HFpEF criteria at follow-up, compared to only 7% in the medical control group [41].

More recent data extend this hemodynamic concept to the right heart pulmonary circulation axis. Hirose et al. showed that about 20% of patients with AFib, preserved LVEF and no prior heart failure had abnormal RV-pulmonary artery (PA) coupling assessed by TAPSE/PA systolic pressure (PASP). Catheter ablation improved TAPSE/PASP at 6 months and reduced the prevalence of RV-PA uncoupling, but this benefit was confined to patients without recurrent AFib. These findings suggest that maintenance of sinus rhythm improves not only left-sided filling and rhythm regularity, but also RV adaption to pulmonary vascular load [42].

Importantly, improved hemodynamics after catheter ablation may also have therapeutic consequences as restoration of sinus rhythm may facilitate initiation and up-titration of guideline-directed heart failure medication. This is clinically relevant because the co-existence of AFib and LVSD can lead to low output, congestion and hypotension, which limits the use of recommended guideline-directed medical therapy (GDMT) for heart failure. As adherence to optimal medical therapy is associated with better outcome in patients with AFib and heart failure regardless of LVEF [43], rhythm control should also be achieved to improve implementation of heart failure therapy.

## Phenotyping of Atrial Fibrillation-induced Arrhythmia-induced Cardiomyopathy and Consequences for Clinical Practice

### Introduction of a New Phenotyping Classification

Although AIC is increasingly recognized in daily clinical practice, a standardized phenotyping system for AFib-related LVSD has not yet been established. As mentioned previously, contemporary terminology follows the concept of distinguishing between “pure” and “superimposed” forms of AIC [6]. However, LV dysfunction caused by AFib is likely more heterogeneous than this binary distinction suggests. AFib may directly cause LV dysfunction in healthy individuals, unmask an occult myocardial substrate, coexist with HFrEF without substantially contributing to further LV impairment, or worsen pre-existing HFrEF. We therefore propose four clinically relevant phenotypes:

The first phenotype comprises patients without preexisting structural heart disease who develop LV dysfunction primarily as a consequence of excessive AFib burden. This corresponds most closely to “pure” AIC. Patients with this phenotype were included in an AIC trial from our group in which 50 patients with tachyarrhythmia (mainly AFib) and newly diagnosed LVSD underwent thorough diagnostic workup including coronary angiography and cardiac MRI to exclude other causes for HFrEF than the arrhythmia itself. 82% of patients fulfilled AIC criteria 6 months after rhythm restoration, with LVEF improving from 35.4% to 57.2% [9]. These data illustrate the most reversible end of the AIC spectrum, in which effective rhythm control regularly leads to (near)-complete recovery of LV function. Nevertheless, even in these apparently “pure” cases, a degree of uncertainty remains, as occult myocardial disease, genetic predisposition, or other markers of myocardial vulnerability may have been present but remained undetected despite comprehensive diagnostic evaluation.

Therefore, the second phenotype consists of patients with occult cardiomyopathy or myocardial vulnerability in whom AFib unmasks LV dysfunction. This includes patients with subclinical fibrosis, impaired strain, prior myocarditis without pronounced clinical symptoms or genetic predisposition. Even after apparent recovery from AFib-induced AIC, many patients show persistent abnormal findings. In a 2024 study, 34 of 41 patients showed recovered LVEF after catheter ablation, but still 58.8% had impaired global LV longitudinal strain, 52.9% had elevated NT-proBNP levels and 29.4% had no improvement in functional capacity [44]. Results from genetic analyses reveal that early-onset AFib cohorts have an enrichment of pathogenic or likely pathogenic variants, predominantly in cardiomyopathy-associated genes. More recent precision medicine data further demonstrated that such variants are found in up to 20% of patients with AFib diagnosed before 60 years and are associated with markers of ventricular substrate, such as elevated T1 and LGE on cardiac MRI [45, 46]. Together, these findings point to the hypothesis that in some patients, AFib represents the first clinical manifestation of an inherited or concealed cardiomyopathic substrate rather than an isolated rhythm disorder.

The third phenotype includes patients with established HFrEF in whom AFib is present but does not substantially worsen LV systolic function. This group is clinically important but poorly defined because most studies are not designed to characterize AFib as a bystander in pre-existing HFrEF. In the above-mentioned AIC trial from our group, 9 of 50 patients were classified as having “non-AIC”. Their LVEF increased from 37.0% to 44.0%, which is well below the established AIC thresholds, demonstrating that AFib is not the dominant cause of LV dysfunction [9].

The fourth phenotype contains patients with pre-existing HFrEF in whom AFib leads to additional deterioration of LV function. This phenotype resembles the “superimposed” form of AIC. It is particularly of interest because structural heart disease does not exclude a clinically meaningful AFib-mediated component of LV dysfunction. This phenotype is commonly encountered in routine clinical practice, where patients with established HFrEF or HFmrEF frequently present with heart failure decompensation precipitated by new-onset or recurrent AFib. Among the proposed phenotypes, patients in this group have very likely been studied most intensively compared to the three other groups in several randomized rhythm control trials, in which patients with established heart failure and concomitant AFib rather than patients with strictly proven “pure” AIC were enrolled [11, 12, 33, 47].

### Towards Prospective Phenotype Assignment

Prospective assignment of these phenotypes will likely require integration of several complementary tools. Cardiac MRI could help identify ventricular fibrosis, while circulating biomarkers such as NT-proBNP may indicate persistent myocardial stress despite apparent clinical improvement. In selected patients, particularly those with early-onset AFib or otherwise unexplained disease, genetic testing could reveal an inherited cardiomyopathic predisposition. In addition, AFib burden, arrhythmia duration and early response to rhythm control may provide further clinically relevant information and together, these parameters may facilitate prospective classification into the four proposed phenotyping categories before complete LVEF recovery has occurred.

### Therapeutical Impact

This phenotype classification has direct clinical implications. In patients with first, second and fourth phenotype, catheter ablation is expected to improve LVEF and those with these phenotypes should undergo interventional rhythm restoration. In contrast, in patients with HFrEF and “bystander” AFib, catheter ablation may not lead to a substantial LVEF recovery. However, this distinction should not be used as the only criterion for or against ablation, because rhythm control provides benefits beyond myocardial recovery, including symptom relief, reduced AFib burden and better quality of life [48, 49].

The phenotypes may be even more important when considering long-term heart failure therapy after successful rhythm control. In WITHDRAW-AF, a highly selected population with presumed AIC, normalized LVEF, stable sinus rhythm for 6 months, low NT-proBNP and absence of LV LGE underwent staged withdrawal of heart failure medication. Most patients maintained LVEF≥50% after withdrawal, supporting the possibility that heart failure drugs can be cautiously discontinued in selected patients under close surveillance [50]. Other data point in a similar direction. In a multicenter cohort of patients with AIC and recovered LVEF after ablation, withdrawal of at least 50% of GDMT did not compromise LVEF, LV dimensions or sinus rhythm maintenance compared with continued therapy, which supports the concept of individualized de-escalation in carefully selected patients [51]. However, this must be balanced against observational data from a broader AIC population, where continuation of renin angiotensin-system inhibitors and beta blockers was associated with a lower risk of heart failure relapse after LVEF improvement [52].

Taken together, medication withdrawal seems to be the most feasible option in phenotype 1: patients who were previously healthy, have no evidence of myocardial disease, maintain sinus rhythm and show complete recovery. In phenotype 2, withdrawal may have negative effects as AFib unmasks an occult cardiomyopathy, genetic predisposition or residual myocardial vulnerability. In phenotype 3, heart failure medication should be guided by the underlying cardiomyopathy rather than by AFib status alone. As the underlying HFrEF substrate persist even if AFib ablation improves LVEF, GDMT should usually be continued in phenotype 4. Importantly, the optimal use, continuation, or withdrawal of guideline-directed medical therapy in patients with concomitant AFib and heart failure remains an area of active investigation and has recently been comprehensively reviewed in our consensus statement, which highlights the limited evidence base and calls for dedicated prospective studies in this frequently encountered patient population [53].

**Fig. 3:**
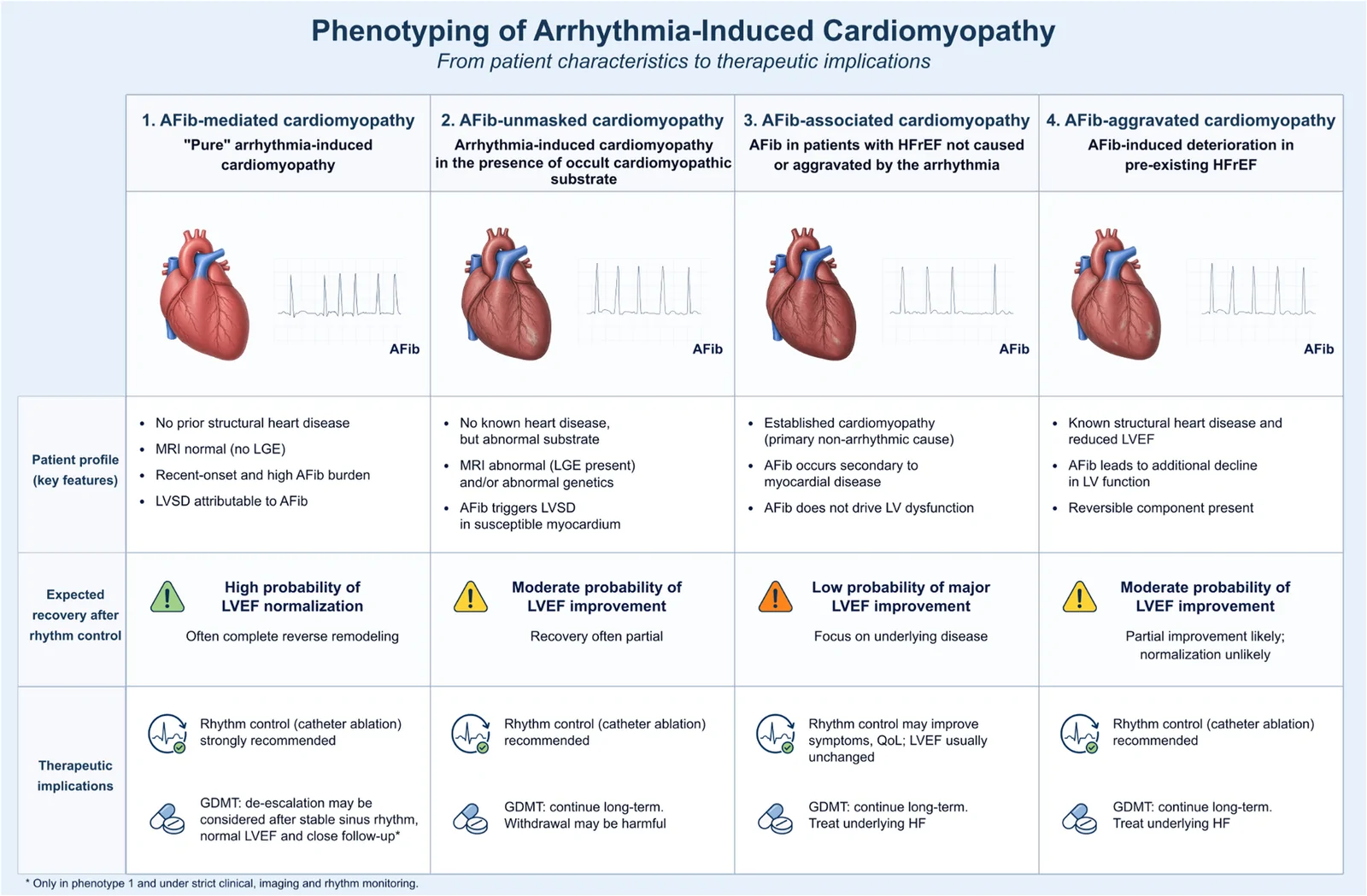
Proposal for a new phenotyping classification of AIC. The effect of AFib on LV function depends on a possible underlying myocardial vulnerability and the individual patient’s response to arrhythmia burden. These four phenotypes highlight important differences in reversibility of HFrEF, rhythm control response and long-term heart failure management. AFib=atrial fibrillation, AIC=arrhythmia-induced cardiomyopathy, GDMT= guideline-directed medical therapy, HFrEF=heart failure with reduced ejection fraction, LGE=late gadolinium enhancement, LV=left ventricular, LVEF=left ventricular ejection fraction, LVSD=left ventricular systolic dysfunction

Phenotyping may help to separate patients in whom rhythm control permits cautious heart failure therapy de-escalation from those in whom lifelong GDMT remains essential (Fig. 3). However, this framework requires rigorous validation in large prospective patient cohorts. The interplay between AFib burden, myocardial substrate, ventricular response, and reversibility of LV dysfunction is highly complex, and patients with AFib and heart failure have not been sufficiently characterized in previous studies to allow reliable phenotype assignment.

## Conclusion

AIC has evolved from the classical concept of tachycardia-induced ventricular dysfunction into a broader heart failure phenotype. Experimental models have established that myocardial structure and function is impaired through several different mechanisms. Translational evidence from human myocardium indicates that remodeling involves altered calcium handling and oxidative stress.

Clinically, AIC remains a retrospective diagnosis, which is confirmed after improvement of LV function. Catheter ablation is the key therapeutic strategy in patients with AFib and heart failure. A new phenotype approach helps to separate different AIC phenotypes according to patient characteristics and individual response to AFib. Future studies should prospectively validate such phenotypes using imaging, rhythm monitoring, genetic testing and biomarkers to guide patient-specific treatment.

TK received travel grants from Boehringer Ingelheim, Lily and Bayer. SS received speakers/consulting honoraria from Astra Zeneca, Novartis, Berlin-Chemie, Bristol-Myers-Squibb, Boehringer Ingelheim, Lilly, Bayer, Pfizer, Siemens Healthineers, Johnson & Johnson, Alnylam. JR has no conflicts of interest to declare.

## Key References

- Shoureshi P, Tan AY, Koneru J, Ellenbogen KA, Kaszala K, Huizar JF. Arrhythmia-Induced Cardiomyopathy: JACC State-of-the-Art Review. J Am Coll Cardiol 2024; 83(22):2214–32
  - ○ The review summarizes key facts about AIC, including pathophysiological mechanisms and underlying arrhythmias
- Prabhu S, Taylor AJ, Costello BT, Kaye DM, McLellan AJA, Voskoboinik A et al. Catheter Ablation Versus Medical Rate Control in Atrial Fibrillation and Systolic Dysfunction: The CAMERA-MRI Study. J Am Coll Cardiol 2017; 70(16):1949–61
  - ○ This randomized trial showed that catheter ablation led to a significantly greater LVEF recovery compared with medical therapy in patients with AFib and unexplained LVSD
- Marrouche NF, Brachmann J, Andresen D, Siebels J, Boersma L, Jordaens L et al. Catheter Ablation for Atrial Fibrillation with Heart Failure. N Engl J Med 2018; 378(5):417–27
  - ○ The randomized CASTLE-AF trial demonstrated the superiority of catheter ablation in heart failure patients with AFib by reducing mortality and heart failure hospitalizations
- Pabel S, Knierim M, Stehle T, Alebrand F, Paulus M, Sieme M et al. Effects of Atrial Fibrillation on the Human Ventricle. Circ Res 2022; 130(7):994–1010
  - ○ Even normofrequent AFib causes distinct cellular remodeling in human LV tissue
- van Gelder IC, Rienstra M, Bunting KV, Casado-Arroyo R, Caso V, Crijns HJGM et al. 2024 ESC Guidelines for the management of atrial fibrillation developed in collaboration with the European Association for Cardio-Thoracic Surgery (EACTS). Eur Heart J 2024; 45(36):3314–414
  - ○ The 2024 ESC guidelines recommend catheter ablation for AFib in patients with HFrEF and suspected AIC
- Sohns C, Fox H, Marrouche NF, Crijns HJGM, Costard-Jaeckle A, Bergau L et al. Catheter Ablation in End-Stage Heart Failure with Atrial Fibrillation. N Engl J Med 2023; 389(15):1380–9
  - ○ The paper extended the benefits of AFib ablation to patients with end-stage heart failure eligible for heart transplantation
- Martens P, Augusto SN, Erzeel J, Pison L, Mullens W, Tang WHW. Effects of Atrial Fibrillation Ablation for Heart Failure With Preserved Ejection Fraction: Insights From CABANA. JACC Heart Fail 2025; 13(5):785–94
  - ○ The study highlights the importance of rhythm restoration in populations with AFib and HFpEF
- Segan L, Kistler PM, Nanayakkara S, Taylor A, Hare J, Costello B et al. Withdrawal of heart failure therapy after atrial fibrillation rhythm control with ejection fraction normalization: the WITHDRAW-AF trial. Eur Heart J 2026; 47(2):250–62
  - ○ This randomized study showed that in carefully selected patients with complete recovery of LV function after catheter ablation, withdrawal of heart failure medication seems tolerable

## Data Availability

No datasets were generated or analysed during the current study.

## Declarations

### AI

The figures in the review were created using Affinity. In addition, some of the small pictograms were designed with AI tools. No permission is needed to use the figures.
